# Optimal trajectory of the neuroendoscope for third ventricle pavement access

**DOI:** 10.3389/fnana.2025.1431128

**Published:** 2025-01-22

**Authors:** Joana Sousa, Susana Maria Silva, Hélio Alves, Bruno Carvalho, José Maria Sousa, Manuel J. Ferreira-Pinto, José Paulo Andrade

**Affiliations:** ^1^Unit of Anatomy, Faculty of Medicine, Department of Biomedicine, University of Porto, Porto, Portugal; ^2^CINTESIS@RISE, Porto, Portugal; ^3^Faculty of Medicine, Department of Clinical Neurosciences and Mental Health, University of Porto, Porto, Portugal; ^4^Department of Neurosurgery, Centro Hospitalar e Universitário de São João, Porto, Portugal; ^5^Department of Neuroradiology, Centro Hospitalar e Universitário de São João, Porto, Portugal; ^6^Movement Disorders and Functional Neurosurgery Unit, Centro Hospitalar e Universitário de São João, Porto, Portugal; ^7^Faculty of Medicine, Department of Surgery and Physiology, University of Porto, Porto, Portugal

**Keywords:** third ventricle, neuroanatomy, endoscopic third ventriculostomy, hydrocephalus, neurosurgery

## Abstract

**Background and aim:**

Endoscopic Third Ventriculostomy (ETV) is used to treat hydrocephalus, an abnormal cerebrospinal fluid accumulation in brain ventricles. By defining a new trajectory and entry point interval, we aim to establish a standardized approach for FreeHand ETV, a vital technique when specialized tools are unavailable, or during emergencies.

**Methods:**

187 MRIs were analyzed, with 30 having hydrocephalus. A pathway crossing the cranial bone, interventricular foramen (of Monro) and tuber cinereum was outlined. Measurements involved distances to cranial sutures, pathway angles and depths, and distances to important anatomical landmarks. Comparisons between hydrocephalic and non-hydrocephalic patients were made while assessing variations linked to age, sex and Evan’s index.

**Results:**

Significant differences were found, notably for depth (93.520 ± 7.228 mm), coronal plane angulation (10.982° ± 6.119°), distance to the sagittal suture (18.957 ± 8.608 mm), and distance to the superior frontal sulcus (7.00 mm). Other variables did not differ significantly between groups, including for the sagittal plane angulation (2.549° ± 3.576°) and the distances to the precentral sulcus (19.93 ± 7.955 mm), and to the coronal suture (10.55 mm).

**Conclusion:**

The new approach, situated close to cranial sutures and distant to the precentral and superior frontal sulcus, shows promise in enhancing surgical precision and outcomes for hydrocephalus management.

## 1 Introduction

Hydrocephalus is a neurological condition characterized by an abnormal cerebrospinal fluid (CSF) accumulation in the brain ventricles, leading to increased intracranial pressure. The clinical presentation of hydrocephalus varies widely across different age groups, presenting significant diagnostic and therapeutic challenges for healthcare professionals worldwide (Koleva and De Jesus, 2024; Yadav et al., 2012). This condition can be caused by various factors, including congenital anomalies, acquired diseases, and trauma, making it a complex disorder that requires individualized management strategies (Koleva and De Jesus, 2024; Yadav et al., 2012). Understanding the underlying cause of hydrocephalus is crucial for its effective treatment.

Obstructive hydrocephalus is one of the most common form of hydrocephalus, occurring when CSF flows within the ventricular system is blocked by an obstruction. This obstruction can result from congenital anomalies, including aqueductal stenosis and Chiari malformations or from acquired factors such as neoplasms (e.g., pineal region tumors, colloid cysts, and posterior fossa tumors), infectious affecting the central nervous system (e.g., meningitis and ventriculitis), and intraventricular hemorrhage (Turner, 2022; Yadav et al., 2012). The management of this conditions is dependent on the precise identification of the obstruction which may require advanced imaging techniques and tailored treatment approaches for each individual case (Yadav et al., 2012).

Normal pressure hydrocephalus (NPH) is another form of hydrocephalus that presents with symptoms such as gait disturbance, cognitive impairment, and urinary incontinence (Ishida et al., 2023; Nieto-Salazar, 2023). Unlike obstructive hydrocephalus, the underlying causes of NPH are multifactorial and not entirely understood, though neurodegenerative diseases and idiopathic alterations in arachnoid villi have been identified as contributing factors. In contrast to obstructive hydrocephalus, endoscopic third ventriculostomy (ETV) might not be one of the primary treatment options for NPH (Hakim et al., 2021; Ishida et al., 2023; Nieto-Salazar, 2023). As an alternative, NPH may be managed with other therapeutic interventions, including ventriculoperitoneal shunting, though the exact treatment approach remains an area of ongoing research.

ETV is an important treatment option for obstructive hydrocephalus, particularly in urgent care settings where rapid interventions is needed (Al-Tamimi et al., 2008; Morgenstern et al., 2011; Yadav et al., 2012). The procedure involves the establishment of an artificial shunt connecting the floor of the third ventricle to the prepontine cistern, allowing the CSF to bypass the obstructed areas and restore normal fluid flow (Jallo et al., 2005). The creation of this pathway is achieved through the insertion of a catheter using endoscopic techniques. The procedure begins with the surgeon carefully creating an initial burr hole at a designated entry point, usually in the frontal bone, and then inserting a rigid or flexible endoscope with a camera on its tip to visualize the brain structures (Jallo et al., 2005). The direction of the catheter should be toward the intersection point between a sagittal plane crossing the ipsilateral medial canthus and a coronal plane crossing 1 cm anterior to the tragus (Jallo et al., 2005). Upon reaching the anterior horn of the lateral ventricle, successful placement within the lateral ventricle is confirmed by observing the visible free flow of CSF from the external tip of the catheter confirms (Morone et al., 2020). Achieving this path may be particularly difficult to envision, mainly due to the three-dimensional nature of the description of the angulation, requiring, therefore, a high level of expertise (Lind et al., 2008). After reaching the interventricular foramen (of Monro), the mammillary bodies and infundibular recess should be identified in the attenuated floor (Jallo et al., 2005). The catheter proceeds into the third ventricle at a proper insertion depth of approximately 9 cm (Jallo et al., 2005), perforating its floor to enable CSF to flow into the pontine cistern. Any deviation from this pattern indicates potential misplacement within the brain parenchyma, requiring catheter redirection until it pierces the walls of the lateral ventricle (Jallo et al., 2005). Typically, this procedure is performed on the right hemisphere, as it is the most frequent non-dominant hemisphere in humans (Morone et al., 2020). Although when the right lateral ventricle morphology is compromised due to vascular and neoplasm pathologies such as hemorrhages and brain tumors, or previous surgical interventions, the left-sided approach offers an alternative route for CSF drainage or intracranial pressure monitoring (Mostofi and Khouzani, 2016).

Stereotactic guidance, MRI, and other imaging techniques can assist in guiding this procedure, especially in complex cases where the anatomy is difficult to navigate (Thomale et al., 2013; Thomale et al., 2018; Watanabe et al., 1987). Nonetheless, the availability of resources in the emergency room or time constraints might limit the use of these technologies in urgent care settings and, in such circumstances, Free-Hand ETV technique is commonly employed, which relies on anatomical landmarks for the most suitable and safe catheter entry point and endoscope inclination (O’Neill et al., 2008). One of the most frequently used landmarks for catheter insertion is the Kocher’s point, located in the frontal bone, which is placed at the midpupillary line, varying from 10 to 12 cm superior and posterior to the nasion, 2 to 3 cm lateral to the sagittal suture, and 1 to 3 cm anterior to the coronal suture (Aref et al., 2017; Morone et al., 2020; Woo et al., 2013). Additionally, differences in skull size, shape, and individual anatomy can impact its precise location and increase the risk of complications such as misplacement or failure to perforate the ventricles (Aref et al., 2017). Mispositioning of the catheter also occurs in up to 45% of cases in some reports (Lane and Akbari, 2022). Factors related to sex, age, and ethnicity can influence anatomical variations in cranial size and geometry influence the risk of catheter misplacement (Howells, 1973; Shah and Koirala, 2014). Notably, lateral ventricle size can vary significantly among individuals, both in cases of hydrocephalus and in those with structurally normal brains (Park et al., 2016).

The lack of standardized coordinates for this reference point has been associated with unperforated ventricles, along with various complications, including hemorrhage, infection, neurological injury due to fornix traction and compression, optic nerve damage, and basilar artery rupture (O’Leary et al., 2000), make it a significant medical condition that requires careful attention from healthcare professionals (Ujjan et al., 2022). As a result, continuous research efforts are crucial to enhance our understanding of the hydrocephalus mechanisms and enhance treatment outcomes. Considering its prevalence and the severity of its consequences, addressing hydrocephalus requires a concerted effort to advance our knowledge and develop more effective therapeutic strategies. This study seeks to establish a standardized and safe approach for Free-Hand ETV by defining a new pathway and proposing a novel entrance interval for the neuroendoscope.

## 2 Materials and methods

3D CISS and T2 SPACE Cranioencephalic MRIs were selected from the SClínico Hospitalar, SPMS© of Centro Hospitalar Universitário de São João (CHUSJ), a tertiary hospital in the Northern Region of Portugal. The study was approved by the Ethical Committee of the Centro Hospitalar Universitário São João/Faculty of Medicine of the University of Porto (no. 228/24). Patients were classified according to hydrocephalus status as hydrocephalic or non-hydrocephalic, based on diagnosis or suspicions written on the electronic medical records by Neurology, Neuroradiology and Neurosurgery specialists.

All patients with preoperative hydrocephalus were included in the study, regardless of its cause. Postoperative MRIs were excluded, as were scans from patients with conditions such as bone fractures, expansive or deforming lesions of the brain parenchyma or meninges, prior brain surgery or radiotherapy, compressive vascular lesions, hemorrhages distorting normal brain anatomy, or congenital brain or cranial deformities, such as Chiari malformation type 1. MRI scans acquired from August 2020 to October 2023 that met the inclusion criteria were selected.

Infratentorial lesions that did not cause any compression or distortion of the supratentorial parenchyma, pituitary agenesis, or non-compressive pituitary lesions were included in the control group, as long as they did not significantly affect the brain anatomy (parenchymal and ventricular disposition) (Guerreiro Stucklin and Grotzer, 2018). Age-related cortex atrophy and ventricular enlargement were not considered exclusion criteria, as these changes are common in hydrocephalus patients, who tend to be older. Patients with any vascular disease were included in the control group unless significant compression or gross anatomical distortion were detected. Meningeal lesions were included unless they caused compression or distortion of the brain parenchyma. Patients with psychiatric or movement disorders did not warrant exclusion.

**Figure 1:**
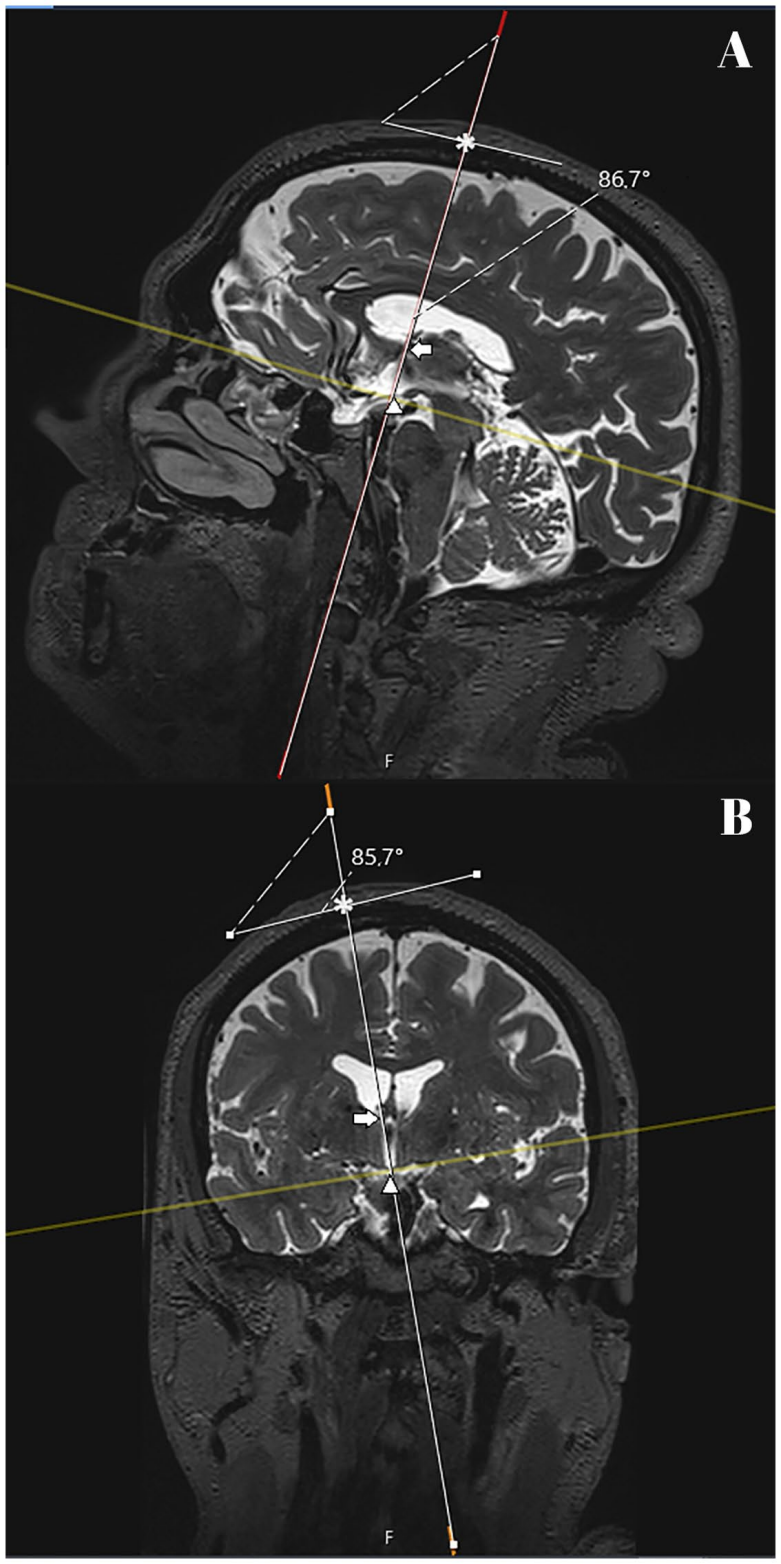
T2 SPACE MRI imaging showing sagittal **(A)** and coronal **(B)** views of the brain hemispheres of a non-hydrocephalic patient with Parkinson’s disease. The full white line represents the delineated pathway of the neuroendoscope for each of the patients. A tangential plane to the entry point was drawn and intersected with the pathway line, marking the upper limit of the pathway (*). The arrow marks the point of intersection of the pathway with the interventricular foramen (of Monro). The tip of the arrowhead marks the intersection between the pathway line and its lower limit, the tuber cinereum. The angulation between the plane tangential to the entry point and the pathway was measured to verify the proximity of the angle to 90° in each patient. The dashed line between the intersecting full lines indicates the side at which the angle was measured. In this patient, the anterior angulation was 86.7° in the sagittal plane **(A)** and the lateral was 85.7° in the coronal plane.

**Figure 2:**
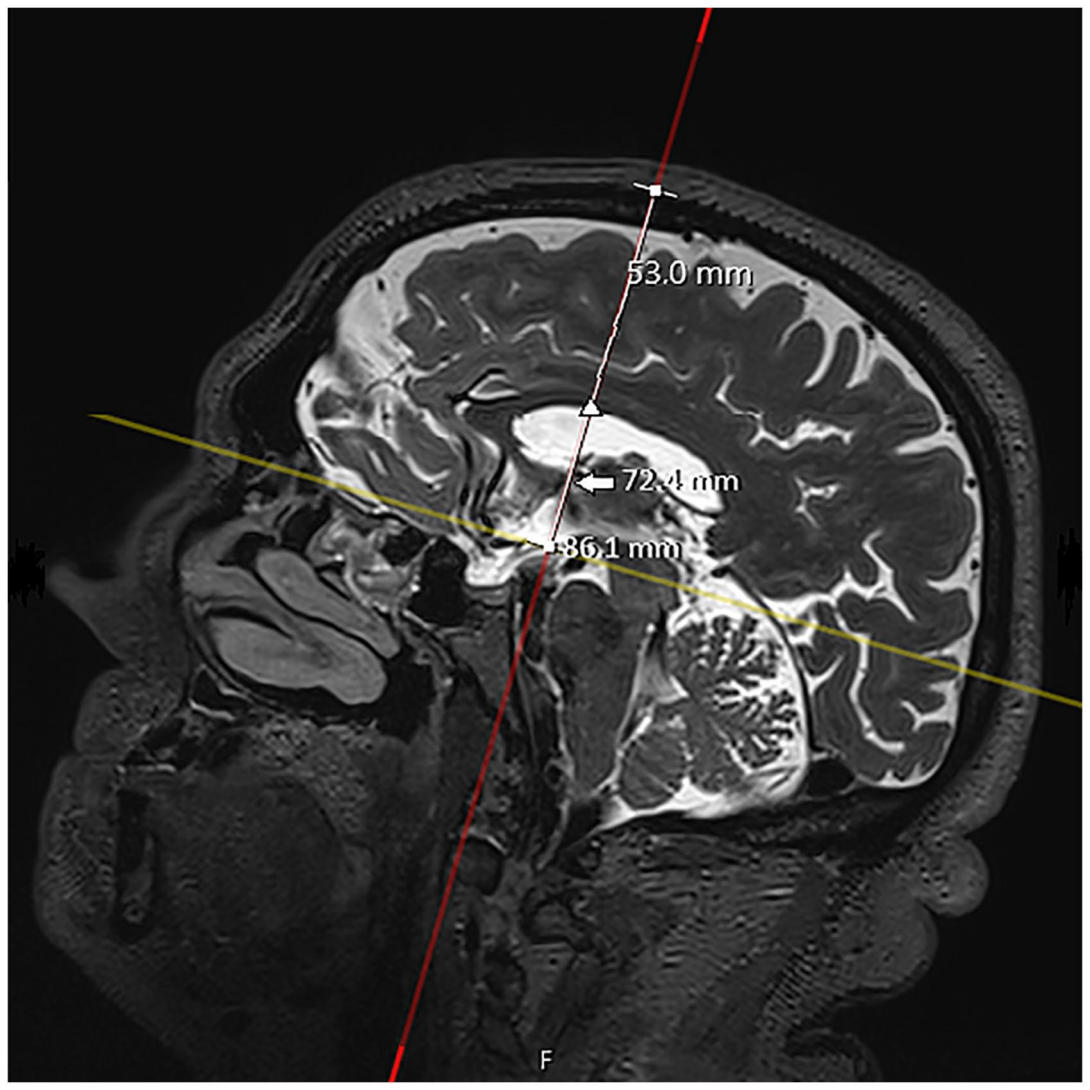
T2 SPACE MRI imaging showing a sagittal view of the brain hemisphere of a non-hydrocephalic patient with Parkinson’s disease. The full white line represents the delineated pathway of the neuroendoscope. The distances between the outer bone lamina (upper square) and the pathway intersections with the tuber cinereum (lower square), interventricular foramen (of Monro) (arrow), and lateral ventricle (arrowhead) were measured. In this patient, the measured depths were 86.1 mm, 72.4 mm, and 53.0 mm, respectively.

Demographic data (gender and age) and MRI indications were documented for each patient; along with their hydrocephalus status. All measurements were performed by a single investigator and exclusively performed on the right hemisphere since it is typically the primary entrance side during ETV procedures to avoid the dominant hemisphere. A reference line was drawn through the interventricular foramen (of Monro), extending from the outer lamina of the skull bone to the tuber cinereum (Figure 1). To minimize any variation due to the brain and skull’s three-dimensional nature, each line was drawn at an angle as close to 90° with the cranial bone as possible. This line represented the expected trajectory of the neuroendoscope during surgery. The required depth of the neuroendoscope was measured along this line, and distances to key landmarks, including distances from the bone to the interventricular foramen (of Monro), and to the intersection of the pathway with the lateral ventricle wall (Figure 2). These measurements are valuable references in clinical practice for anticipating key milestones during procedures, including both the depth required to reach the interventricular foramen (of Monro) and in providing insight into when to expect the onset of free flow of CSF through the catheter insertion in the lateral ventricle in hydrocephalus patients (Ludwig et al., 2022).

**Figure 3:**
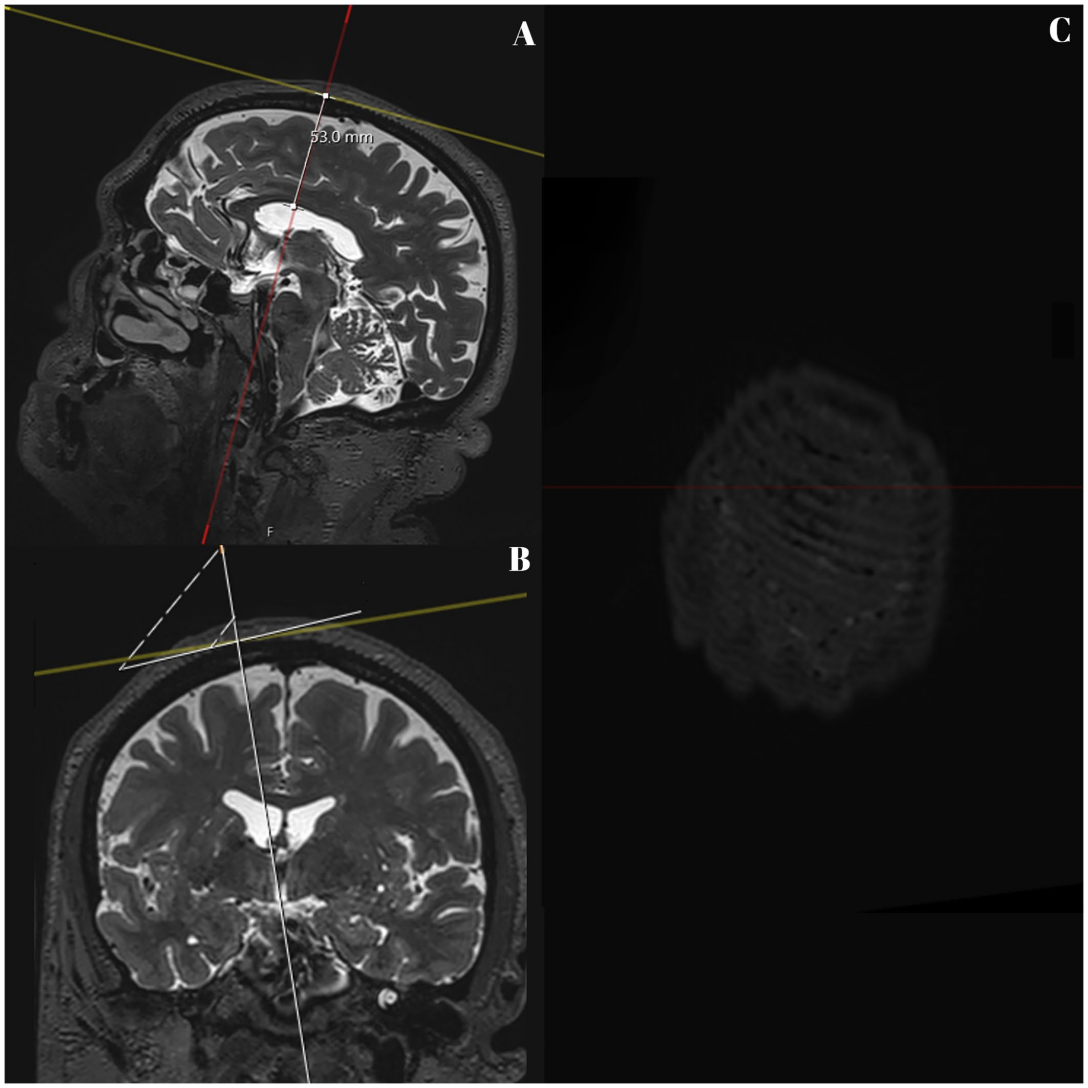
T2 SPACE MRI imaging showing sagittal **(A)** and coronal **(B)** views of the brain hemispheres, and transversal **(C)** view of the skull of a non-hydrocephalic patient with Parkinson’s disease. The full white line represents part of the delineated pathway of the neuroendoscope, and the depth up to the lateral ventricle wall is still shown (53.0 mm). The upper square marks the entry point on the outer bone lamina in **A** and **B**. A line was drawn perpendicular to the pathway and passed through the entry point (white square), as seen in **A** and **B**. **C** shows the image as seen from above.

**Figure 4:**
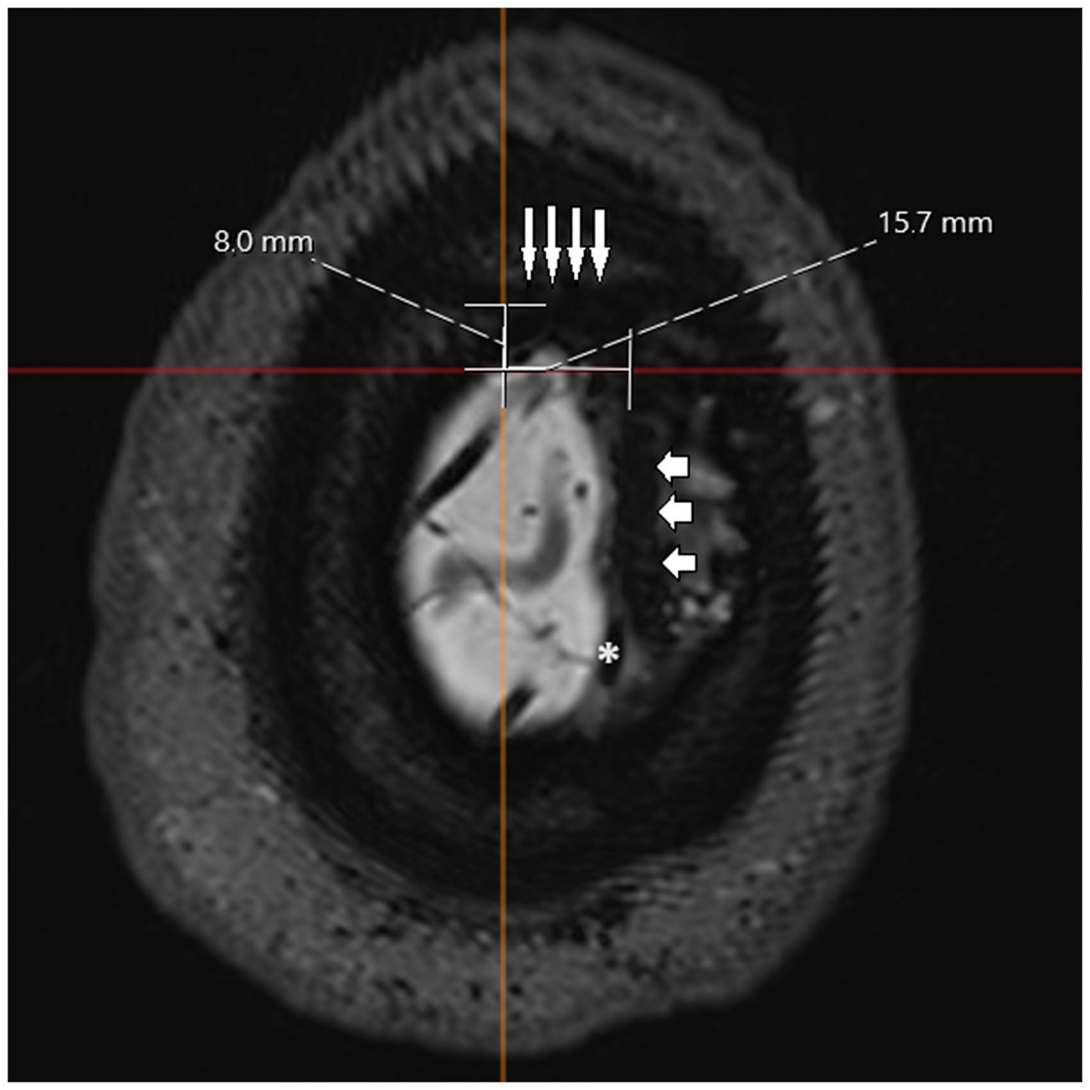
T2 SPACE MRI imaging showing a transversal view of the brain hemispheres and part of the skull of a non-hydrocephalic patient with Parkinson’s disease. The sagittal suture is marked with short, horizontal arrows, and the coronal suture is marked with long, vertical arrows. Due to the nature of the plane used, it is impossible to show the entire skull with both sutures at the same time, and part of the brain hemispheres are visible. The sagittal suture can be seen overlapping the superior sagittal sinus (marked as *). The longest horizontal and vertical lines were drawn to intersect at the established entry point. From this intersection, the perpendicular distance was measured relative to the coronal suture (vertical white line) and the sagittal suture (horizontal white line). In this case, the measured distances were 8.0 and 15.7 mm, respectively.

Entry point coordinates were assessed through a tangential transversal plane (Figure 3) by measuring distances from the coronal and sagittal sutures to the entry point by following two perpendicular lines that intersected at the location point (Figure 4). Negative values indicated entry points positioned anterior to the coronal suture or laterally to the left of the sagittal suture. Safe distances to the motor cortex and superior frontal gyrus were also assessed by measuring the perpendicular distance between the surgical pathway and the precentral sulcus (on the sagittal plane) and superior frontal sulcus (on the coronal plane). Depending on the variable considered, negative values indicated the pathway was positioned anterior to the precentral sulcus and lateral to the superior frontal sulcus. Angulations and distances were analyzed using Brainlab Elements software (version 6.0, Brainlab AG©) for the sagittal and coronal planes and Sectra Workstation IDS7 software (version 24.1.10.5437, Sectra AB©) for other measurements. Angulation was measured relative to the anterior commissure-posterior commissure plane (AC-PC plane), where positive values indicated a direction toward the posterior half of the skull in the sagittal plane, and negative values indicated an anterior direction. Similarly, negative values in the coronal plane indicated an inclination toward the left half of the skull.

**Figure 5:**
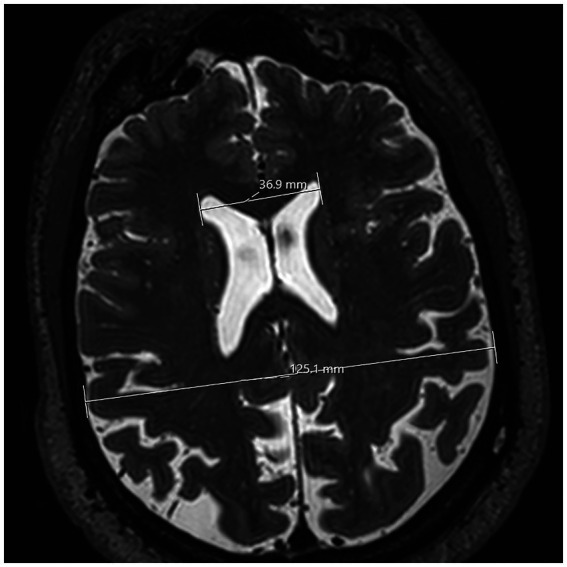
T2 SPACE MRI imaging showing a transversal view of the brain hemispheres of a non-hydrocephalic patient with Parkinson’s disease. The plane for this section is horizontal. Both lateral ventricles can be viewed. The lateral extremities of the upper white line mark the greatest distance between the anterior horns of the lateral ventricle. The lower white line’s lateral extremities mark the inner maximum diameter of the skull. In this case, the measurements of each line were 36.9 mm and 125.1 mm, respectively. These measurements were then used to calculate Evan’s Index.

These measurements, referred to as “surgical parameters,” are essential for defining a safe and effective approach to ETV. Additionally, the maximum width of the anterior horns of the lateral ventricles and the maximum diameter of the inner skull were measured (Figure 5). The Evan’s Index, defined as the ratio of the maximal width of the frontal horns to the maximum inner skull diameter, was used to assess ventricular size in NPH patients, serving as a valuable tool for standardizing ventricle size relative to the skull size in individuals (Howells, 1973).

To account for age-related brain degeneration, typically after the age of 50 to 60 years (a decrease of gray matter and a rate of increase of the brain ventricles), though in some cases degenerative process starts earlier (somewhere between 30 and 40 years of age) (Kaye et al., 1992; Peters, 2006), patients were divided into two groups: those aged 50 years or younger (to include early degenerative changes) and those older than 50 (to capture more advanced changes). This cutoff was chosen to maintain a balanced sample size across both groups.

Statistical analysis was done using SPSS (IBM Corp. Released 2022. IBM SPSS Statistics for Windows, Version 29.0. Armonk, NY: IBM Corp), with a significance level set at *α* = 0.05. Descriptive statistics, including means and standard deviations (or medians and 25th and 75th percentiles, as appropriate), were presented for continuous variables, while categorical variables were presented as absolute counts and proportions. Differences between the hydrocephalic and control groups (demographic variables and surgical parameters) were compared using independent samples t-tests (or the non-parametric Mann–Whitney-*U* test) and the chi-squared test, as appropriate.

In the hydrocephalus group, Pearson’s correlation (or Spearman’s rank correlation, according to the distribution of the variables) were used to explore associations between the different surgical parameters. Multivariate linear regression models, including sex (female being the reference category), age (≤ 50 years as the reference category), and Evan’s Index (as a continuous variable) as covariates, were fitted for each surgical parameter to adjust for potential confounders. Considering the lack of relevant associations in univariate or bivariate correlations analysis, linear regression model was not performed for variables with highly skewed distribution (e.g., distances to the coronal suture or to the superior frontal sulcus), and correlations were used instead.

## 3 Results

A total of 187 MRIs were selected for analysis. Within this dataset, 157 MRIs (84%) were of patients exhibiting no discernible structural abnormalities within the hemispheres, ventricular system, or skull. The remaining 30 MRIs (16%) were of patients presenting with hydrocephalus (see Table 1).

**Table 1:** Descriptive data of the overall studied sample.

Variables		Patients with hydrocephalus (*n* = 30; 16.0%)			Patients without hydrocephalus (*n* = 157; 84.0%)			*p*-value of the Chi-Squared test
		*n*	M	SD	*n*	M	SD	
Age in years (*M* = 56.05; SD = 14.588)	≤50 years (*n* = 51; 27.3%)	8 (15.7%)	57.77	17.853	43 (27%)	55.72	13.921	0.935
	>50 years (*n* = 136; 72.7%)	22 (16.2%)			114 (72.6%)			
Evan’s Index (*M* = 0.280; SD = 0.0545)	Evan’s Index <0.3 (*n* = 148; 79.1%;)	3 (10.0%)	0.375	0.070	145 (92.4%)	0.262	0.024	<0.001
	Evan’s Index ≥0.3 (*n* = 39; 20.9%)	27 (90.0%)			12 (7.6%)			
Sex	Female (*n* = 98; 52.4%)	15 (15.3%)						0.773
	Male (*n* = 89; 47.6)	15 (16.9%)						

*n, number; M, mean; SD, standard deviation; EI, Evan’s Index.*

Seven of the selected MRIs were acquired using CISS sequencing, and the remaining 181 were obtained using T2 SPACE weighting. Four out of the 187 MRIs could not be accessed through the Brainlab Elements software, preventing the identification of the distance to the precentral and superior frontal sulci. Consequently, four data entries were missing for these variables, with one belonging to a hydrocephalic patient.

### 3.1 Demographic information

Of the selected patients, 98 (52.4%) were women and 89 (47.6%) were men, of which 15 women (15.3%) and 15 men (16.9%) were previously diagnosed with hydrocephaly. The mean age of the overall studied population was 56.05 years, with a standard deviation of 14.588 years. The ages of the included patients ranged from 19 to 87 years of age. There were no statistically significant differences in the distribution of hydrocephalic and non-hydrocephalic patients as far as sex (*p* = 0.773) and age categories (*p* = 0.935) are concerned (see Table 1).

### 3.2 Underlying pathologies

The different underlying pathologies of patients in the control group are listed in Table 2. Among these patients, a significant portion (55.4%) were patients with severe Parkinson’s disease which underwent preoperative MRI scans for Deep Brain Stimulation. The remaining 44.6% encompassed patients with diverse vascular pathologies (like arteriovenous malformations or vascular aneurisms), as well as neoplastic (namely cystic pituitary gland or other disorders of this gland, among others), psychiatric (including obsessive-compulsive disorder and dementia), and neurological disorders, including other non-parkinsonian movement disorders.

**Table 2:** Underlying pathologies in hydrocephalic and non-hydrocephalic patients.

Pathology/Syndrome	Number of non-hydrocephalic patients	Number of hydrocephalic patients
Benign and malignant tumors	31	11
Movement disorders	89	-
Vascular disease	16	-
Psychiatric disorders	7	-
Normal pressure hydrocephalus	-	11
Other neurological disorders	14	8
Total	157	30

In contrast, patients diagnosed with hydrocephalus exhibited mainly brain tumors as the primary cause, with some cases attributed to normal pressure hydrocephalus (see Table 2). For discriminated underlying pathologies for both groups, consult the supplementary material.

### 3.3 Depth up to the tuber cinereum

For hydrocephalus patients, the mean depth of the neuroendoscope for hydrocephalus patients (mean = 93.520 mm, standard deviation = 7.228 mm) is significantly larger than that of control group patients (mean = 88.543 mm, standard deviation = 3.949, *p* = 0.001). Cohen’s *d* of −1.077 denotes a large effect size, indicating a substantial difference between groups. The range of depth for the studied hydrocephalic patients is between 81.6 and 108.9 mm (see Table 3).

**Table 3:** Comparison of surgical parameters between non-hydrocephalic and hydrocephalic patients.

Variable	Overall sample (*n* = 187)	Patients without hydrocephaly (*n* = 157)	Patients with hydrocephaly (*n* = 30)	Range	*p*-value	Cohen’s *d*
Depth up to the tuber cinereum, mm	M = 89.341; SD = 4.958	M = 88.543; SD = 3.949	M = 93.520; SD = 7.228	81.6; 108.9	0.001	−1.077
Depth up to the interventricular foramen, mm	M = 72.938; SD = 4.250	M = 72.590; SD = 3.812	M = 74.763; SD = 5.794	65.2; 89.2	0.056	−0.519
Depth up to the lateral ventricle wall, mm	M = 51.124; SD = 7.512	M = 53.482; SD = 4.812	M = 38.787; SD = 7.057	15.2; 82.7	<0.001	2.811
Angulation in the sagittal plane, degrees	M = 2.287; SD = 3.417	M = 2.237; SD = 3.396	M = 2.549; SD = 3.576	−10.5; 10.6	0.662	−0.091
Angulation in the coronal plane, degrees	M = 8.057; SD = 4.237	M = 7.498; SD = 3.533	M = 10.982; SD = 6.119	0.9; 25.5	0.005	−0.860
Distance of the entry point to the sagittal suture, mm	M = 15.408; SD = 6.288	M = 14.730; SD = 5.521	M = 18.957; SD = 8.608	2.8; 42.0	0.014	−0.692
Distance of the entry point to the coronal suture, mm	MD = 8.400; p25 = 4.700 p75 = 13.300	MD = 8.00; p25 = 4.80; p75 = 12.90	MD = 10.55; p25 = 4.10; p75 = 15.10	0.00; 32.1	0.456	0.109
Distance to the precentral sulcus, mm	M = 18.230; SD = 7.453	M = 17.92; SD = 7.339	M = 19.93; SD = 7.955	0.00; 39.00	0.213	−0.271
Distance to the superior frontal sulcus, mm	MD = 10.00; p25 = 5.00; p75 = 14.00	MD = 10.00; p25 = 6.00; p75 = 15.00	MD = 7.00; p25 = 0.00; p75 = 11.50	−18.00; 22.00	0.014	0.370

*n, number of patients; M, median; SD, standard deviation; MD, median; p25, 25th percentile value; p75, 75th percentile value.*

### 3.4 Depth up to the ventricle wall

The mean depth up to the lateral ventricle wall for hydrocephalus patients is 38.787 mm, suggesting a notably shorter average distance (*p* < 0.001) than for the control group (mean = 53.482 mm). However, the broader standard deviation (standard deviation = 7.057 mm) indicates a considerable increase in variation around the mean, which might be explained by the individual differences in the deformities of the normal brain anatomy according to the severity of the hydrocephalus (see Table 3).

### 3.5 Distance to the sagittal suture

There are also significant differences (*p* = 0.014) in mean perpendicular distances to the sagittal suture between patients with hydrocephalus (mean = 18.957 mm; standard deviation = 8.608 mm) and patients from the control group (mean = 14.730 mm; standard deviation = 5.521 mm), whose mean is notably smaller. The minimum and maximum measured distances were 2.8 mm and 42.0 mm. Cohen’s d of −0.692 denotes a medium effect size between groups. However, a lateral deviation of approximately 4 mm in the entry point, as seen by the difference between these means, could be considered noticeable in clinical practice.

### 3.6 Distance to the superior frontal sulcus

The median distances to the superior frontal sulcus are also significantly different (*p* = 0.014) between hydrocephalus patients (median = 7.00 mm) and the control group (median = 10.00 mm). However, Cohen’s d is 0.370, suggesting this variable has a small effect size between groups. A quarter of patients has a distance of 0 mm to the superior frontal sulcus or a negative value in these variables (as defined per convention in the methods section), suggesting either direct adjacency to this landmark or a lateral position to the sulcus. In contrast, 75% of patients have a distance of 11.5 mm or less to the superior frontal sulcus (see Table 3).

### 3.7 Coronal plane angulation

As for the average angulation measured in the coronal plane of the pathway, there are statistically significant differences between means (*p* = 0.005). Although Cohen’s d indicates a large effect size of this variable (*d* = 0.860), in clinical practice, the difference between the estimated mean for hydrocephalic patients (mean = 10.982°; standard deviation = 6.119°) and the control group (mean = 7.498°; standard deviation = 3.533°) will most likely not be distinguishable to the naked eye. The angulation ranges from 0.9° to 25.5° in hydrocephalic patients, and a protractor may be required during the procedure (see Table 3).

The remaining variables under examination did not demonstrate statistically significant differences between the groups, as indicated by *p*-values greater than 0.05.

### 3.8 Sagittal plane angulation

Hydrocephalic patients display a broad spectrum of sagittal plane angulations, spanning from −10.5° to 10.6°, with variations centering around the value 0. The mean angulation for hydrocephalic patients (mean = 2.549°) and non-hydrocephalic patients (mean = 2.237°) were closely aligned, suggesting a prevalent posterior inclination in both groups. Consequently, the orientation of the neuroendoscope can vary, allowing for either anterior or posterior inclination based on the specific characteristics of each patient (see Table 3).

The study reveals notable findings, including a significant positive correlation with the coronal angulation (*p* = 0.016, *r* = 0.437, see Table 4). This suggests that as the endoscope becomes more horizontal in the coronal plane, its alignment in the sagittal plane also tends toward a horizontal orientation.

**Table 4:** Correlation between surgical parameters in patients presenting with hydrocephalus.

	Variable	Correlation coefficient	*p*-value
Distance to the precentral sulcus, mm	Distance to the sagittal suture, mm	−0.461	0.012
	Distance to the coronal suture, mm	−0.124	0.521
	Depth up to the lateral ventricle wall, mm	0.322	0.088
	Sagittal plane angulation, degrees	−0.372	0.047
	Coronal plane angulation, degrees	−0.154	0.426
	Evan’s Index	−0.408	0.028
Distance to the sagittal suture, mm	Distance to the coronal suture, mm	0.256	0.173
	Evan’s Index	−0.430	0.018
Sagittal plane angulation, degrees	Coronal plane angulation, degrees	0.437	0.016
	Evan’s Index	0.287	0.124
Coronal plane angulation, degrees	Evan’s Index	0.109	0.568

### 3.9 Distance to the coronal suture

Across the hydrocephalic group, the median distance from the entry point to the coronal suture is 10.55 mm. No statistical differences were noted between hydrocephalic and non-hydrocephalic groups. The entry point was quite close to the coronal suture in most patients, with 50% of the patients presenting a distance to this suture between 4.10 mm and 15.10 mm. Most entry points lie within a distance of about 15 mm from the cranial suture, with only a quarter of them surpassing this value (see Table 3).

Furthermore, the entry point distances toward the coronal and sagittal sutures did not exhibit a significant relationship between themselves (*p* = 0.173), indicating that, although one might increase, the other might not change accordingly (see Table 4).

### 3.10 Distance to the precentral sulcus

The computed range for the hydrocephalic patients was 0.00 to 39.00 mm. Attention should be focused on the fact that the minimum value of this range is not negative, indicating that the pathway does not extend anterior to this sulcus in any of these patients (see Table 3).

Surprisingly, a moderately negative correlation exists between the distance to the precentral sulcus and the entry point distance to the sagittal suture (*r* = −0.461*, p* = 0.012). This finding suggests that as the distance to the sagittal suture increases, a corresponding decrease occurs in the distance to the precentral sulcus. Conversely, no significant association was found for the distance to the coronal suture (*p = 0.521*), indicating that alterations in one distance do not necessarily influence the other.

Consequently, an indirect relationship between the distances toward the precentral sulcus and the sagittal suture might be at play, with adjustments in coronal and sagittal plane angulations serving as intermediate steps in this complex relationship. Not so surprisingly, a statistically significant association, although weak, is present between the precentral sulcus and the sagittal plane angulation (*r* = −0.372*, p* = 0.047). As for the depth up to the lateral ventricle wall, no association is present (*p* = 0.088), meaning that a deformity of the brain parenchyma severe enough to reduce this distance significantly does not result in a change in the distance to the precentral sulcus. As for the coronal plane angulation, no association exists with the distance to the precentral sulcus (*p* = 0.426). For correlations with the distance to the precentral sulcus, refer to Table 4.

### 3.11 Evan’s index

There was a statistically significant difference in the distribution of patients with and without hydrocephalus between each Evan’s Index category (*p* < 0.001). Evan’s Index for hydrocephalus patients (mean = 0.375; standard deviation = 0.070) and for the control group (mean = 0.262; standard deviation = 0.024). Nevertheless, three of these patients did not cross this cut-off, despite the diagnosis, as opposed to 12 non-hydrocephalic patients who crossed over it (see Table 1).

The sagittal and coronal angulations did not correlate significantly with Evan’s Index (*p* = 0.124 and *p* = 0.568, respectively). However, a notable finding emerged concerning the distance toward the precentral sulcus, which displayed a moderately negative association with Evan’s Index (*r* = −0.408; *p* = 0.028). This negative association implies that in individuals with larger ventricles relative to the maximum skull diameter, albeit not as markedly enlarged in absolute terms compared to others with broader skulls, there is a reduction in the distance to the precentral sulcus. Consequently, a higher risk of the perforation of the motor cortex might be at stake.

Furthermore, a significant moderately positive association between the distance of the entry point to the sagittal suture and Evan’s Index is present (*r* = 0.430*, p* = 0.018), indicating that the entry point deviates laterally as the size of the lateral ventricles increases.

For correlations with Evan’s Index, refer to Table 4.

### 3.12 Multiple linear regression models

Multiple linear regression models were employed to examine the influence of various factors on several surgical parameters. Notably, for the depth of the neuroendoscope, significant associations were observed with both age (*p* = 0.027) and Evan’s Index (*p* = 0.001), while sex showed no statistically significant relationship (*p* = 0.322). This suggests that age is a significant determinant, as individuals aged over 50 years tend to have, on average, shallower depths by 5.905 mm compared to their younger counterparts, even after adjusting for sex and Evan’s Index. Additionally, Evan’s Index exhibited a notable independent effect, indicating that for every 0.1 increase in Evan’s Index, the depth of the neuroendoscope increased on average by 5.8911 mm.

Similarly, when investigating the distance to the interventricular foramen (of Monro), a significant relationship with Evan’s Index was found after adjusting for potential confounding effects from age and sex (*p* = 0.003), indicating an average increase of 4.409 mm for every 0.1 unit of Evan’s Index. However, neither sex nor age significantly affected this particular surgical parameter (*p* = 0.178 and *p* = 0.138, respectively).

Conversely, concerning pathway angulations, a borderline significant association was found between sex and sagittal plane angulation (*p* = 0.042), suggesting that males may exhibit a more acute posterior angle than females, with an angle 2.383° smaller, on average. However, no similar relationship could be extrapolated for the coronal plane angulation (*p* = 0.370). Age also exerted a significant effect on the posterior angulation (*p* = 0.006) and coronal plane angulation (*p* = 0.020), with patients aged over 50 years displaying increases of 3.784° in the sagittal plane angulation and increases of 6.032° in coronal plane angulation, when compared with their younger counterparts. Evan’s Index did not exhibit a significant association with either angle (*p* = 0.226 and *p* = 0.876, respectively), emphasizing the distinct influence of patient demographics on these anatomical measurements.

Lastly, for the distances between the entry point and cranial sutures, Evan’s Index emerged as a significant predictor for the distance to the sagittal suture (*p* = 0.027), and the distance to the precentral sulcus (*p* = 0.037). A mean increase of 5.073 mm in the sagittal suture and a decrease of 4.561 in the distance toward the precentral sulcus was demonstrated for each increase of 0.1 units in Evan’s Index. As for sex and age, no statistically significant association was found with the distance to the sagittal suture (*p* = 0.518 and *p* = 0.555, respectively) and the distance to the precentral sulcus (*p* = 0.537 and *p* = 0.987, respectively).

## 4 Discussion

The following section provides a comprehensive analysis and interpretation of the gathered data from the present research to shed light on relevant risks and considerations of the established novel pathway and respective entry point interval for ETV in urgent care of hydrocephaly.

We also explore the interplay between several surgical parameters and demographic data of the sample, including age and sex, as well as the relative size of the lateral ventricles. The synthesis of these findings not only elucidates the intricate dynamics at play, but also might provide valuable insights into patient-oriented decision-making.

### 4.1 Methodology

A potential bias in the hydrocephalus group arises from excluding postoperative hydrocephalus patients, as the severity and individual characteristics of hydrocephalus can differ significantly between preoperative and post-operative contexts. However, since most ETV patients typically undergo preoperative brain imaging, the MRIs obtained from CHUSJ system likely represent a comprehensive subset of individuals, mitigating bias to some extent by the uniformity in preoperative imaging practices among hydrocephalus patients undergoing ETV.

The control group selection, based on MRIs from patients with suspected underlying pathologies other than hydrocephalus might not entirely reflect the general population. To address this, exclusion criteria were applied to eliminate pathologies causing significant deformation of brain and ventricle anatomy. However, this group may still overrepresent older and more ill individuals, as young and healthier people are less prone to need brain MRIs. Nevertheless, these differences should not be interpreted as bias, as hydrocephalus primarily affects older people. Given the intra-hospital context and retrospective nature of this study, selecting individuals without any pathology is unlikely.

Strengthening the conclusions, the sample encompassed all hydrocephalus cases regardless of underlying etiology, acknowledging that treatment modalities can vary based on the specific type of hydrocephalus. While NPH is typically managed with a ventriculoperitoneal, ventriculoatrial or lumboperitoneal shunt rather than an ETV (Hakim et al., 2021; Ishida et al., 2023; Nieto-Salazar, 2023), some studies have already shown positive results for ETV management in patients with normal pressure hydrocephalus (Komlakh et al., 2022; Komlakh et al., 2023). ETV is also indicated for obstructive hydrocephalus, including cases involving tumors and hemorrhage, though the primary treatment for tumors usually involves excision rather than ETV (Yadav et al., 2012). Nonetheless, ETV remains crucial in life-threatening situations, or when delayed surgical scheduling is necessary.

As a result, not all cases in the present study were primary candidates for ETV. Our focus was to evaluate surgical parameters in the presence of anatomical distortion caused by enlarged ventricles. Therefore, our findings remain pertinent for understanding the impact of ventricular size during the procedure, despite the heterogeneity in hydrocephalus etiologies within our sample.

Finally, a single-investigator analysis might also be seen as a drawback in this study. Separate measurements conducted by multiple investigators, with subsequent calculation of the mean for each parameter, might yield more accurate results.

### 4.2 Baseline data

Besides age and sex, variation in skull morphology, namely due to anatomical differences between ethnicities, might have a role in variations of Evan’s Index and surgical parameters (Fleseriu and Karavitaki, 2018; Howells, 1973; Komlakh et al., 2022; Techataweewan et al., 2018). Age-associated disparities were assessed by categorizing patients according to a cutoff of 50 years. Statistical analysis revealed no significant difference in age distribution between hydrocephalic and non-hydrocephalic patients.

The age distribution among non-hydrocephalic patients skews toward individuals aged over 50 years. This phenomenon arises from the lower frequency of MRI scans performed among younger individuals for diagnostic purposes due to lesser susceptibility to central nervous system disorders or disorders of other nature compared with older individuals. Consequently, in this sample, individuals without hydrocephalus may exhibit higher Evan’s Index values compared with the general population due to age-related atrophy of the brain (Kaye et al., 1992; Komlakh et al., 2023; Peters, 2006).

Therefore, it is crucial to acknowledge that these findings might not fully represent the broader population, as the studied sample might potentially overrepresent older people.

However, considering our primary focus on hydrocephaly treatment, which predominantly affects older individuals (Abdelmowla and Essa, 2019; Edwards et al., 2004; Isaacs et al., 2018), comparing the hydrocephalus sample with an older population is vital for drawing valid comparisons, given that no statistical significance between age categories is present.

### 4.3 Depth up to the tuber cinereum and depth up to the ventricle wall

The neuroendoscope depth to the tuber cinereum in the studied hydrocephalic patients ranged from 8 to 11 cm, while the mean depth to the ventricular wall was approximately 4 cm, significantly different from non-hydrocephalic patients (see Table 3). An important association was found between the depth to the tuber cinereum and Evan’s Index, even after adjusting for age and sex variables (see Table 5). For every 0.1 unit increase in Evan’s Index, the depth tends to increase by approximately 6 mm, while the distance to the ventricular wall decreases by about 7 mm. This inverse proportion relationship may allow surgeons to predict the depth at which the neuroendoscope tip perforates the tuber cinereum according to the penetration depth at which CSF starts to flow, allowing intraoperative adjustment of their approach and avoiding over or underpenetration during the procedure. Moreover, individuals over 50 years tend to exhibit, on average, a shorter depth up to the tuber cinereum, even after accounting for sex and Evan’s Index differences, with a mean decrease of about 6 mm.

**Table 5:** Multiple linear regression models for different surgical variables, according to age, sex, and Evan’s Index.

Dependent variable	Predictors	Adjusted regression coefficient (IC 95%)	*p*-value	Dependent variable	Predictors	Adjusted regression coefficient (IC 95%)	*p*-value
Depth up to the tuber cinereum, mm [*F*(3,26) = 5.607, *p* = 0.004, *R*^2^ = 0.39]	Sex	2.220 (−2.302; 6.742)	0.322	Coronal plane angulation, degrees (*F*(3,26) = 2.254, *p* = 0.106, R^2^ = 0.21)	Sex	−1.943 (−6.139; 2.433)	0.370
	Age	−5.905 (−11.096; −0.715)	0.027		Age	6.032 (1.009; 11.055)	0.020
	Evan’s Index	5.891 (2.605; 9.177)	0.001		Evan’s Index	0.244 (−2.937; 3.424)	0.876
Depth up to the interventricular foramen (of Monro), mm [F(3,26) = 4.281, *p* = 0.014, *R*^2^ = 0.33]	Sex	2.561 (−1.245; 6.367)	0.178	Distance to the sagittal suture, mm (F(3,26) = 2.349, *p* = 0.096, R^2^ = 0.21)	Sex	1.955 (−4.175; 8.084)	0.518
	Age	−3.252 (−7.620; 1.117)	0.138		Age	2.048 (−4.988; 9.085)	0.555
	Evan’s Index	4.409 (1.643; 7.174)	0.003		Evan’s Index	5.073 (0.619; 9.528)	0.027
Depth up to the lateral ventricle wall, in mm [F(3,26) = 9.404, *p* < 0.001, *R*^2^ = 0.52]	Sex	1.176 (−2.747; 5.100)	0.543	Distance to the precentral sulcus, mm (F(3,26) = 1.823, *p* = 0.169, R^2^ = 0.18)	Sex	1.788 (−4.102; 7.679)	0.537
	Age	−0.837 (−5.341; 3.667)	0.706		Age	−0.053 (−6.759; 6.654)	0.987
	Evan’s Index	−7.075 (−9.927; −4.224)	<0.001		Evan’s Index	−4.561 (−8.817; −0.305)	0.037
Sagittal plane angulation, degrees [F(3,26) = 5.023, *p* = 0.007, *R*^2^ = 0.37]	Sex	−2.383 (−4.667; −0.098)	0.042				
	Age	3.784 (1.161; 6.406)	0.006				
	Evan’s Index	1.003 (−0.657; 2.663)	0.226				

Reference categories were age ≤ 50 years and female sex; Evan’s Index was included in the model as a continuous variable with its value divided by 10, so the coefficients can be interpreted as a 0.1 units increase in Evan’s Index.

Considerable variations between minimum and maximum values of the distance to the lateral ventricle wall (approximately 1.5 cm and 8 cm, respectively) reflect the diverse patterns of anatomy distortion due to hydrocephalus. Therefore, further studies are necessary to refine and establish a narrower distance interval for the initial free flow of CSF. Such refinements will further enhance its relationship with Evan’s Index and its predictive value regarding the depth to reach the tuber cinereum, increasing surgical precision for already promising results.

Jallo et al. (2005) describe a distance of approximately 9 cm from the dura mater to the floor of the third ventricle when inserting the endoscope 3 cm lateral to the sagittal suture and above the coronal suture. Despite variations in entry points, this distance overlaps with the mean depth of 93.520 mm. In contrast, Morone et al. (2020) and Woo et al. (2013) suggest a 6 cm depth from Kocher’s point. While this shorter distance may seem advantageous in reducing the amount of brain parenchyma perforated, this approach demands significant spatial orientation from the surgeon, as the description of the angulation and entry point may prove challenging to visualize, as elaborated upon in the subsequent discussion section (Morone et al., 2020).

### 4.4 Depth up to the interventricular foramen (of Monro)

The variation of the depth to the tuber cinereum is accompanied by a variation of the distance to the interventricular foramen (of Monro), with a range of approximately 6.5 to 9 cm and a mean of 7.5 cm. This suggests that surgeons can anticipate the crossing of the interventricular foramen (of Monro) at around 7.5 cm, offering a somewhat predictable landmark during the procedure for enhanced surgical planning, albeit its evident wide range. However, individual anatomical variations and underlying pathology may still affect the precise depth reached during surgery. In patients with hydrocephalus, an average increase of about 4 mm occurs for each increase in 0.1 units of Evan’s Index, after adjusting for sex and age (see Table 5). Thus, while the consistency in reaching the interventricular foramen (of Monro) might be promising, surgeons must remain adaptable to subtle intraoperative nuances to ensure optimal outcomes.

The mean for non-hydrocephalic patients was about 7 cm. No statistically significant differences were seen between hydrocephalus groups (see Table 3). This suggests that, despite the development of hydrocephalus increasing the depth up to the tuber cinereum compared to a structurally normal brain, the interventricular foramen (of Monro) is as distant, on average, as for non-hydrocephalic patients.

### 4.5 Pathway angulation

The sagittal plane angulation in hydrocephalic patients ranged from −10.5° to 10.6°, averaging at about 2.5°, and highlights notable variation in the angulation of the endoscope. Therefore, a difference of 22° in anteroposterior inclination is present between measured extremes, corresponding roughly to 11° on each side (see Table 3). It is worth noting that the sagittal angulation tends to exhibit a posterior deviation with age, with patients over 50 years showing an increase of about 4° (see Table 5). Since the range centers around 0° with a slight posterior inclination among the sample, aligning the neuroendoscope with a perpendicular line passing through the AC-PC plane, with a minor posterior adjustment may facilitate the procedure. Hence, considering an age-related trend in hydrocephalus (Isaacs et al., 2018), it is advisable to prioritize a slight posterior inclination in initial attempts. Further adjustments to the direction of the neuroendoscope might be required if failure to reach the pavement of the third ventricle is apparent.

Although preoperative assessment of the AC-PC plane may pose a drawback, patients typically undergo prior imaging for diagnostic purposes, eliminating the need for additional scans. Alternatively, rapid imaging techniques like cranioencephalic computed tomography scan can swiftly evaluate the AC-PC plane in cases where pre-existing images are unavailable.

In the coronal plane, hydrocephalic patients exhibited a wide range of angulation (1° to 26°, with an average of 11°), differing significantly from non-hydrocephalic patients, contrary to the sagittal plane angulation (see Table 3). Arguably, while the observed range of angulation may appear large for both the side-to-side and anteroposterior inclinations, the range and the differences between groups reflect the complex interplay of anatomical, clinical, and surgical factors inherent in endoscopic procedures and the underlying pathology.

As established by a positive moderate Pearson’s correlation coefficient, if there is any non-desirable deviation of the coronal plane angulation, slight modifications of the sagittal angle may be required, and vice-versa (see Table 4). Similarly to the sagittal plane angulation, the coronal plane angulation showed age-related variations, with individuals over 50 exhibiting approximately 6° more lateral tilt when compared to younger individuals, accompanied by a slight increase of 4° in the sagittal angulation. Both angulations, they also increase with age. However, interestingly, neither sagittal nor coronal angulations were significantly associated with Evan’s Index, suggesting that the size and deformation of the ventricles does not influence the tilt angle of the endoscope during the procedure unless other factors, such as older age, are at play (see Table 5). While there is a small notable difference in sagittal plane angulation between sexes, with a seemingly negative effect for women (*r* = −2.383), the reliability of this correlation in our study may be compromised, as seen by its borderline *p*-value (*p* = 0.042). Therefore, further studies with a larger sample of hydrocephalic patients are warranted to compare the association between sexes and anteroposterior angulation.

Regarding a Kocher’s point approach, surgeons should aim for a perpendicular angle to the intersection of lines drawn from the ipsilateral medial canthus and external auditory meatus (Morone et al., 2020). However, achieving this precise angle presents challenges during the procedure (Abdoh et al., 2012). Surgeons may encounter difficulty in accurately visualizing and executing the perpendicular angle, potentially resulting in errors in catheter placement (Abdoh et al., 2012). Moreover, instrument manipulation within confined spaces is often necessary to achieve the desired angle, further limiting visibility during the procedure and increasing the risk of inaccurate placement (Morone et al., 2020). These challenges underscore the complexity of using Kocher’s point as a reference for catheter insertion, highlighting the need for careful consideration and expertise in ventriculostomy procedures (Morone et al., 2020).

The superficial angulation, in this case, was guaranteed to be as close to 90° as possible. As such, this concern is anticipated to be minimal in most patients, since no individuals had a superficial angulation inferior to 80° on both planes. Furthermore, the angulation measurements were also taken with respect to the AC-PC plane, considering the potential application of these measurements in ETV procedures with concurrent neuronavigation, a crucial aspect in contemporary surgical techniques.

### 4.6 Entry point coordinates

The entrance point in hydrocephalic patients was determined based on perpendicular distances to the sagittal and coronal sutures, with average coordinates of 2 cm and 1 cm, respectively. The location of the entry point is less than 4.1 mm away from the coronal suture in a quarter of patients, suggesting relative proximity to this suture. Similarly, the 75th percentile reveals that three-fourths of the entry points are positioned within 15.10 mm of the suture (see Table 3). Therefore, most entry points lie within a distance of about 1.5 cm from the coronal suture, with only a quarter of them surpassing this value. This proximity facilitates visual identification by the surgeon. The distance to the sagittal suture varied widely ranging from 2.8 mm to 42.0 mm (see Table 3). No correlation was revealed between the two coordinates of the entry point (see Table 4); indicating that a change in one coordinate does not necessarily correspond to a change in the other.

This variability constitutes a challenge in targeting specific anatomical structures or lesions during the procedure, potentially leading to suboptimal surgical outcomes or necessitating additional adjustments intraoperatively. Additionally, the broad range increases the risk of encountering critical structures or causing accidental damage during endoscope insertion (Lane and Akbari, 2022; O’Leary et al., 2000) and, thus, the precise knowledge of the anatomical area is also fundamental (da Silva et al., 2024; Fernandes-Silva et al., 2021).

Despite these challenges, it should be noted that this variability encompasses the diverse spectrum of anatomical variations and complexities encountered in clinical practice due to hydrocephalus. For instance, a 0.1 unit increase in Evan’s Index leads to an average increase of 5 mm increase in distance from the sagittal suture, indicating that severity of the hydrocephalus status leads to the increase in distance (see Table 5). Further studies are warranted, including investigations into variation in distance according to etiology, to refine and narrow the range of this entrance point and to enhance precision and safety in endoscopic procedures, ultimately improving patient outcomes. Yadav et al. (2012) and Jallo et al. (2005) describe a narrower entry point range, typically 2.5 to 3 cm lateral to the sagittal suture and over or anterior to the coronal suture. However, the maximum measured distance to the sagittal suture was 4 cm, and its lower limit was 3 mm, exceeding the range proposed by those studies. A mean distance to the sagittal suture of 1.8 cm reinforces this proposition. Consequently, a significant part of our patients likely did not have entry points within this specified range.

For the coronal suture, the median distance was 10.55 mm, with half of the hydrocephalic patients having an entry point between 4.10 mm and 15.10 mm away from this landmark. While most points were typically located posterior to the coronal suture, approximately a quarter of the entry points were less than 4 mm, potentially indicating that some entry points were located anteriorly to the coronal suture. However, the minimum value observed was 0 mm, meaning that some entry points were directly over the coronal suture, and no entry point was anterior to it. This contributes to standardizing the procedure, as it suggests that the approach should consistently be posterior to this suture, contrary to what is described in the following works.

The majority of documented entry points for ETV are situated anterior to the coronal suture. Aref et al. (2017) explored a new entry point located 4 cm anterior to the coronal suture. Despite yielding statistically significant and promising outcomes, with 95.4% of patients undergoing a successful ETV procedure and a low associated complication rate (7.6%), the authors caution that the risk of motor cortex injury and associated sequelae due to fornix traction should prompt surgeons to employ neuronavigation when employing this approach, pending further validation through additional studies.

In contrast, Morone et al. (2020) and Woo et al. (2013) describe Kocher’s point as being located at the mid-pupillary line, ranging from 11 cm superior and posterior to the nasion, 3 cm lateral to the sagittal suture, and 1 to 2 cm anterior to the coronal suture. However, Aref et al. (2017) define this point as being 1 cm anterior to the coronal suture and along the mid-pupillary line without further elaboration regarding the sagittal suture. This description is quite difficult to visualize in three dimensions and requires the surgeon to have a good spatial orientation. Besides that, this range does not apply to the entry points measured in the location points of our patients. Other entry points are reported by Morone et al. (2020) but they are not as commonly used during ETV.

### 4.7 Distances to precentral and superior frontal sulci

The distance analysis to the superior frontal sulcus focused on patients with hydrocephalus, as statistically significant differences were observed compared to the control group. The median distance of 7 mm suggests that, on average, the entry points are situated at a small distance from the superior frontal sulcus. This proximity suggests that the entry points are relatively close to this crucial anatomical landmark within the brain. Conversely, the 75th percentile value of 11.5 mm indicates that the majority of entry points are located within a relatively short, although still safe, distance from the superior frontal sulcus. This range suggests that most patients have entry points that are situated close to this sulcus but still with a safe margin to avoid direct interference.

However, the presence of cases where the distance is 0 mm or less at the 25th percentile denotes a closely located or sagittal suture overlapping entry point in a subset of patients. This scenario raises concerns, namely regarding a potentially higher damage to the superior frontal gyrus during the procedure, as further proved by the minimum measured value in hydrocephalic patients of −18 mm, i.e., almost 2 cm lateral to this sulcus (see Table 3).

Damage to the superior frontal gyrus can lead to impairments in cognitive functions associated with the superior frontal gyrus, such as working memory performance, including spatial orientation, manipulation, monitoring, and decision-making (Alagapan et al., 2018; Jurado and Rosselli, 2007). Surgeons, therefore, must carefully assess the potential risks, notably deficits in working memory, and benefits, such as alleviating acute symptoms or preventing further deterioration (Alagapan et al., 2018; Jurado and Rosselli, 2007). Consequently, further studies should be conducted to evaluate the risks associated with this procedure regarding the perforation of this gyrus.

As for the precentral sulcus, the distance for patients presenting with hydrocephalus ranged from 0 to approximately 4 cm (see Table 3). Therefore, the drawn pathway avoids crossing over this sulcus in every hydrocephalic patient of this sample. In some patients, however, the pathway overlaps the sulcus, as shown by a distance of 0 mm. While theoretically, no deficits should be expected from this overlapping, further clinical studies are necessary to validate this association and ascertain the lack of risk of perforating the motor cortex. No significant differences are present in the distance to the precentral sulcus between hydrocephalic and non-hydrocephalic groups. Given that the current range does not encompass negative values, if future studies confirm the safety of this range regarding motor cortex lesions, it could also imply safety for hydrocephalic patients with ventricle anatomy closer to that of a structurally normal brain. A correlation established between the distance to the precentral sulcus and the distance to the sagittal suture suggests that as the distance to the sagittal suture increases, there is a corresponding decrease in the distance to the precentral sulcus (see Table 4). Furthermore, an indirect relationship between the distances toward the precentral sulcus and the sagittal suture might exist, with adjustments in coronal and sagittal plane angulations potentially serving as intermediate steps.

There was no association between the distance from the bone to the lateral ventricle wall and the precentral sulcus, suggesting that significant brain parenchyma deformities do not necessarily affect the distance to the precentral sulcus. At first glance, one might think there is no increased risk of perforation. However, the distance to the precentral sulcus shows a moderately negative association with Evan’s Index (see Table 4). The negative association implies that individuals with larger ventricles relative to the maximum skull diameter have a reduced distance to the precentral sulcus, potentially indicating a higher risk of perforating the motor cortex. This risk might be more significant in shorter people and women, whose skulls tend to be slightly smaller (Biswas et al., 2015), although further studies are needed to better assess this relationship.

### 4.8 Evan’s Index

To assess the impact of ventricle sizes on the measured surgical parameters, Evan’s Index was employed to normalize for variations attributed to variations in skull diameter (Alagapan et al., 2018). Despite its widespread use, Evan’s Index has some limitations (Toma et al., 2011; Zhou and Xia, 2022). One such drawback is the susceptibility of measurements to variations according to imaging planes and angles while assessing the maximum width of the cranium and frontal horns of the lateral ventricles (Toma et al., 2011; Zhou and Xia, 2022). Despite these limitations, Evan’s Index remains the most commonly used ratio for evaluating relative ventricular size, particularly in patients with normal pressure hydrocephalus (Zhou and Xia, 2022).

However, several considerations warrant caution in the interpretation of these findings. Despite the observed statistical significance for differences between Evan’s Index categories, it is essential to acknowledge the presence of overlapping Evan’s Index values between hydrocephalic and non-hydrocephalic patients. Three hydrocephalic patients who fall below the established Evan’s Index threshold of 0.3 were identified, raising questions about the sensitivity of this measure and its ability to capture variability in ventricular size across diverse patient populations (see Table 1). This overlap implies, therefore, that Evan’s Index alone may not be entirely indicative of hydrocephaly in all cases, which goes in line with other studies that defend a comprehensive approach for diagnosis, including incorporating additional clinical data and age and sex-focused cut-offs (Brix et al., 2017; Pradhan et al., 2021; Vudhivanich and Tarathipayakul, 2022; Zhou and Xia, 2022), as well as other alternative Evan’s Index measurement methods (Zhou and Xia, 2022).

Nevertheless, the Chi-Square test offers compelling evidence supporting Evan’s Index as a discriminative factor between individuals with and without hydrocephalus. Moreover, the mean Evan’s Index value for patients with hydrocephalus significantly exceeded that of the control group, reinforcing the association between an Evan’s Index ≥0.3 and the presence of hydrocephalus. Despite its utility, while Evan’s Index offers valuable insights, clinicians should exercise it cautiously and consider supplementary diagnostic modalities alongside Evan’s Index to ensure thorough patient evaluation (Toma et al., 2011).

Twelve out of 157 non-hydrocephalic patients also surpassed the 0.3 threshold, denoting the lower specificity of Evan’s Index (see Table 1). This trend may stem from a combination of factors, including variations in skull morphology and dimensions, albeit the anticipated predominant role of brain atrophy due to the increased age of the studied sample (Markov et al., 2022; Missori et al., 2016; Vudhivanich and Tarathipayakul, 2022).

## 5 Conclusion

This study examines an alternative approach to ETV by focusing on a new entry point, pathway depth, and its relationship with key surgical landmarks. We propose an entry point located posterior to the coronal suture, avoiding the precentral sulcus. This method may reduce the risk of motor cortex lesion and fornix traction compared to anterior entry points, though further studies are necessary to validate its efficacy in clinical practice. Additionally, we highlight the need for additional investigation to confirm potential risks related the superior frontal gyrus damage. Moreover, we ensured that the angle was as close to 90 degrees as possible, streamlining the procedure and potentially enhancing its accuracy and safety when compared with the complex estimation of angulation using Kocher’s point.

In conclusion, we suggest the optimal neuroendocospic trajectory involves an entry point 2 cm lateral to the sagittal suture and 1 cm posterior to the coronal suture, with an angulation with the surface of the cranium between 80° and 90° in both the coronal and sagittal plane. This trajectory maintains a distance of about 7 mm from the superior frontal sulcus and between 0 and 4 cm from the precentral sulcus.

## Data Availability

The raw data supporting the conclusions of this article will be made available by the authors, without undue reservation.

## References

[ref1] Abdelmowla R. A. A. Essa A. A. (2019). Ventriculoperitoneal shunt: impact of nursing management on outcomes of patients with adult hydrocephalus. ARC J. Nurs. Healthc. 5, 9–21. doi: 10.20431/2455-4324.0501002

[ref2] Abdoh M. G. Bekaert O. Hodel J. Diarra S. M. Le Guerinel C. Nseir R.. (2012). Accuracy of external ventricular drainage catheter placement. Acta Neurochir. 154, 153–159. doi: 10.1007/s00701-011-1136-9, PMID: 21892637

[ref3] Alagapan S. Lustenberger C. Hadar E. Shin H. W. Frӧhlich F. (2018). Low-frequency direct cortical stimulation of left superior frontal gyrus enhances working memory performance. NeuroImage 184, 697–706. doi: 10.1016/j.neuroimage.2018.09.064, PMID: 30268847 PMC6240347

[ref4] Al-Tamimi Y. Z. Bhargava D. Surash S. Ramirez R. E. Novegno F. Crimmins D. W.. (2008). Endoscopic biopsy during third ventriculostomy in paediatric pineal region tumours. Childs Nerv. Syst. 24, 1323–1326. doi: 10.1007/s00381-008-0632-6, PMID: 18365207

[ref5] Aref M. Martyniuk A. Nath S. Koziarz A. Badhiwala J. Algird A.. (2017). Endoscopic third Ventriculostomy: outcome analysis of an anterior entry point. World Neurosurg. 104, 554–559. doi: 10.1016/j.wneu.2017.05.052, PMID: 28532915

[ref6] Biswas S. Chowdhuri S. Das A. Mukhopadhyay P. P. (2015). Observations on symmetry and sexual dimorphism from Morphometrics of foramen magnum and orbits in adult Bengali population. J. Indian Acad. Forensic Med. O. 37, 346–351. doi: 10.5958/0974-0848.2015.00090.1

[ref7] Brix M. K. Westman E. Simmons A. Ringstad G. A. Eide P. K. Wagner-Larsen K.. (2017). The Evans’ index revisited: new cut-off levels for use in radiological assessment of ventricular enlargement in the elderly. Eur. J. Radiol. 95, 28–32. doi: 10.1016/j.ejrad.2017.07.013, PMID: 28987681

[ref8] da Silva A. C. Silva S. M. Alves H. Cunha-Cabral D. Pinto F. F. Fernandes-Silva J.. (2024). Stereotactic anatomy of the third ventricle. Surg. Radiol. Anat. 46, 271–283. doi: 10.1007/s00276-024-03312-1, PMID: 38374441 PMC10960742

[ref9] Edwards R. J. Dombrowski S. M. Luciano M. G. Pople I. K. (2004). Chronic hydrocephalus in adults. Brain Pathol. 14, 325–336. doi: 10.1111/j.1750-3639.2004.tb00072.x, PMID: 15446589 PMC8096062

[ref10] Fernandes-Silva J. Silva S. M. Alves H. Andrade J. P. Arantes M. (2021). Neurosurgical anatomy of the floor of the third ventricle and related vascular structures. Surg. Radiol. Anat. 43, 1915–1925. doi: 10.1007/s00276-021-02785-8, PMID: 34128100

[ref11] Fleseriu M. Karavitaki N. (2018). Non-functioning pituitary adenomas, not all the same and certainly not boring! Pituitary 21, 109–110. doi: 10.1007/s11102-018-0875-5

[ref12] Guerreiro Stucklin A. S. Grotzer M. A. (2018). Cerebellar tumors. Handb. Clin. Neurol. 155, 289–299. doi: 10.1016/B978-0-444-64189-2.00019-6, PMID: 29891066

[ref13] Hakim F. Jaramillo-Velásquez D. Gonzalez M. Gómez D. F. Ramón J. F. Serrano-Pinzón M. (2021). “Normal pressure hydrocephalus: revisiting the hydrodynamics of the brain” in Cerebrospinal Fluid. eds. Bektaşoğlu P. K. Gürer B. (London, United Kingdom: IntechOpen Limited), 1–32.

[ref14] Howells W. W. (1973). Cranial variation in man: a study by multivariate analysis of patterns of difference among recent human population. Cambridge, mass. Papers of the Peabody Museum of Archaeology and Ethnology, Harvard University 67, 1–259.

[ref15] Isaacs A. M. Riva-Cambrin J. Yavin D. Hockley A. Pringsheim T. M. Jette N.. (2018). Age-specific global epidemiology of hydrocephalus: systematic review, metanalysis and global birth surveillance. PLoS One 13:e0204926. doi: 10.1371/journal.pone.0204926, PMID: 30273390 PMC6166961

[ref16] Ishida T. Murayama T. Kobayashi S. (2023). Current research of idiopathic normal pressure hydrocephalus: pathogenesis, diagnosis and treatment. World J. Clin. Cases 11, 3706–3713. doi: 10.12998/wjcc.v11.i16.3706, PMID: 37383114 PMC10294169

[ref17] Jallo G. I. Kothbauer K. F. Abbott I. R. (2005). Endoscopic third ventriculostomy. Neurosurg. Focus. 19, E11–E14. doi: 10.3171/foc.2005.19.6.12, PMID: 16398476

[ref18] Jurado M. B. Rosselli M. (2007). The elusive nature of executive functions: a review of our current understanding. Neuropsychol. Rev. 17, 213–233. doi: 10.1007/s11065-007-9040-z, PMID: 17786559

[ref19] Kaye J. A. DeCarli C. Luxenberg J. S. Rapoport S. I. (1992). The significance of age-related enlargement of the cerebral ventricles in healthy men and women measured by quantitative computed X-ray tomography. J. Am. Geriatr. Soc. 40, 225–231. doi: 10.1111/j.1532-5415.1992.tb02073.x, PMID: 1538040

[ref20] Koleva M. De Jesus O. (2024). Hydrocephalus. Treasure Island, Florida: StatPearls Publishing. 32809710

[ref21] Komlakh K. Oveisi H. Aghamiri S. H. (2023). Evaluation of treatment response results in patients with normal-pressure hydrocephalus undergoing surgery. J. Family Med. Prim. Care 12, 101–105. doi: 10.4103/jfmpc.jfmpc_834_22, PMID: 37025224 PMC10071903

[ref22] Komlakh K. Oveisi H. Hossein Aghamiri S. (2022). Endoscopic third ventriculostomy as treatment option for normal pressure hydrocephalus. Eur. J. Transl. Myol. 32:10618. doi: 10.4081/ejtm.2022.10618 36259576 PMC9830389

[ref23] Lane J. Akbari S. H. A. (2022). Failure of endoscopic third Ventriculostomy. Cureus 14:e25136. doi: 10.7759/cureus.25136, PMID: 35733459 PMC9205383

[ref24] Lind C. R. Tsai A. M. Law A. J. Lau H. Muthiah K. (2008). Ventricular catheter trajectories from traditional shunt approaches: a morphometric study in adults with hydrocephalus. J. Neurosurg. 108, 930–933. doi: 10.3171/JNS/2008/108/5/0930, PMID: 18447709

[ref25] Ludwig H. Dreha-Kulaczewski S. Bock C. (2022). CSF upward motion is crucial for ETV success. Preprints 2022, 2022010148. doi: 10.20944/preprints202201.0148.v1 (Accessed, January 14, 2025).

[ref26] Markov N. T. Lindbergh C. A. Staffaroni A. M. Perez K. Stevens M. Nguyen K.. (2022). Age-related brain atrophy is not a homogenous process: different functional brain networks associate differentially with aging and blood factors. Proc. Natl. Acad. Sci. USA 119:e2207181119. doi: 10.1073/pnas.2207181119, PMID: 36459652 PMC9894212

[ref27] Missori P. Rughetti A. Peschillo S. Gualdi G. Di Biasi C. Nofroni I.. (2016). In normal aging ventricular system never attains pathological values of Evans’ index. Oncotarget 7, 11860–11863. doi: 10.18632/oncotarget.7644, PMID: 26919252 PMC4914253

[ref28] Morgenstern P. F. Osbun N. Schwartz T. H. Greenfield J. P. Tsiouris A. J. Souweidane M. M. (2011). Pineal region tumors: an optimal approach for simultaneous endoscopic third ventriculostomy and biopsy. Neurosurg. Focus. 30:E3. doi: 10.3171/2011.2.FOCUS10301, PMID: 21456930

[ref29] Morone P. J. Dewan M. C. Zuckerman S. L. Tubbs R. S. Singer R. J. (2020). Craniometrics and ventricular access: a review of Kocher’s, Kaufman’s, Paine’s, Menovksy’s, Tubbs’, Keen’s, Frazier’s, Dandy’s, and Sanchez’s points. Oper. Neurosurg. 18, 461–469. doi: 10.1093/ons/opz194, PMID: 31420653

[ref30] Mostofi K. Khouzani R. K. (2016). Surface anatomy for implantation of external ventricular drainage: some surgical remarks. Surg. Neurol. Int. 7, 577–S580. doi: 10.4103/2152-7806.189437, PMID: 27625894 PMC5009573

[ref31] Nieto-Salazar M. A. (2023). Normal pressure hydrocephalus: clinical approach and practical considerations. Open access. J. Neurol. Neurosurg. 17:OAJNN.MS.ID.555972. doi: 10.19080/oajnn.2023.17.555972

[ref32] O’Leary S. T. Kole M. K. Hoover D. A. Hysell S. E. Thomas A. Shaffrey C. I. (2000). Efficacy of the Ghajar guide revisited: a prospective study. J. Neurosurg. 92, 801–803. doi: 10.3171/jns.2000.92.5.0801, PMID: 10794294

[ref33] O’Neill B. R. Velez D. A. Braxton E. E. Whiting D. Oh M. Y. (2008). A survey of ventriculostomy and intracranial pressure monitor placement practices. Surg. Neurol. 70, 268–273. doi: 10.1016/j.surneu.2007.05.007, PMID: 18207539

[ref34] Park J. Son W. Park K. S. Kim M. Y. Lee J. (2016). Calvarial slope affecting accuracy of Ghajar guide technique for ventricular catheter placement. J. Neurosurg. 124, 1429–1433. doi: 10.3171/2015.5.JNS15226, PMID: 26544778

[ref35] Peters R. (2006). Ageing and the brain. Postgrad. Med. J. 82, 84–88. doi: 10.1136/pgmj.2005.036665, PMID: 16461469 PMC2596698

[ref36] Pradhan A. Chalise U. Shrestha A. Dhungana S. (2021). Study of Normal values of Evan’s index on brain CT scan in individuals attending Nepal medical college teaching hospital, Kathmandu, Nepal. Nepal Med. College J. 23, 41–47. doi: 10.3126/nmcj.v23i1.36227

[ref37] Shah S. Koirala S., Jha, C-B. (2014). Effect of ethnicity on head form anthropometry of 17-26 year old normal population in eastern Nepal. Eur. J. Anat. 18, 135–139.

[ref38] Techataweewan N. Dudzik B. Kitkhuandee A. Duangthongphon P. Tayles N. (2018). Gender and population variation in Craniometry and Freehand pass Ventriculostomy. World Neurosurg. 117, e194–e203. doi: 10.1016/j.wneu.2018.05.240, PMID: 29890273

[ref39] Thomale U. W. Knitter T. Schaumann A. Ahmadi S. A. Ziegler P. Schulz M.. (2013). Smartphone-assisted guide for the placement of ventricular catheters. Childs Nerv. Syst. 29, 131–139. doi: 10.1007/s00381-012-1943-1, PMID: 23089936

[ref40] Thomale U. W. Schaumann A. Stockhammer F. Giese H. Schuster D. Kästner S.. (2018). GAVCA study: randomized, multicenter trial to evaluate the quality of ventricular catheter placement with a Mobile health assisted guidance technique. Neurosurgery 83, 252–262. doi: 10.1093/neuros/nyx420, PMID: 28973670 PMC6140776

[ref41] Toma A. K. Holl E. Kitchen N. D. Watkins L. D. (2011). Evans’ index revisited: the need for an alternative in Normal pressure hydrocephalus. Neurosurgery 68, 939–944. doi: 10.1227/NEU.0b013e318208f5e0, PMID: 21221031

[ref42] Turner S. (2022). Central nervous system tumors. London: IntechOpen Limited.

[ref43] Ujjan B. U. Nawaz S. Anwer M. S. Rehman S. Kamran M. Hassan N. (2022). Prevalence and pattern of initial complications following endoscopic third Ventriculostomy for hydrocephalus Obstructor. Pakistan J. M H Sci. 16, 570–572. doi: 10.53350/pjmhs20221612570

[ref44] Vudhivanich M. Tarathipayakul T. (2022). Evaluation of the Evans’ index in Thailand population using computed tomography accompany with magnetic resonance imaging. J. Med. Assoc. Thail. 105, 555–559. doi: 10.35755/jmedassocthai.2022.06.13321

[ref45] Watanabe E. Watanabe T. Manaka S. Mayanagi Y. Takakura K. (1987). Three-dimensional digitizer (neuronavigator): new equipment for computed tomography-guided stereotaxic surgery. Surg. Neurol. 27, 543–547. doi: 10.1016/0090-3019(87)90152-2, PMID: 3554569

[ref46] Woo H. Kang D. H. Park J. (2013). Preoperative determination of ventriculostomy trajectory in ventriculoperitoneal shunt surgery using a simple modification of the standard coronal MRI. J. Clin. Neurosci. 20, 1754–1758. doi: 10.1016/j.jocn.2013.01.025, PMID: 24035649

[ref47] Yadav Y. R. Parihar V. Pande S. Namdev H. Agarwal M. (2012). Endoscopic third ventriculostomy. J. Neurosci. Rural Pract. 3, 163–173. doi: 10.4103/0976-3147.98222, PMID: 22865970 PMC3409989

[ref48] Zhou X. Xia J. (2022). Application of Evans index in Normal pressure hydrocephalus patients: a Mini review. Front. Aging Neurosci. 13:783092. doi: 10.3389/fnagi.2021.783092, PMID: 35087391 PMC8787286

